# An Empirical Study on the Factors Influencing the Number of Fans of Female-Oriented Accounts on Chinese Tik Tok

**DOI:** 10.3389/fpsyg.2022.826819

**Published:** 2022-06-23

**Authors:** Yanyan Du, Shengliang Lin, Xueli Zhang

**Affiliations:** ^1^School of Humanities, Zhejiang University of Technology, Hangzhou, China; ^2^School of Journalism and Communication, Jinan University, Guangzhou, China; ^3^Department of Communication, Fujian Normal University, Fuzhou, China

**Keywords:** female-oriented accounts on Chinese TikTok, fans, influential factors, statistics, inner needs

## Abstract

This paper selects 500 samples through a recommendation algorithm and, on the basis of content analysis, examines the factors that affect the number of fans of female-oriented accounts on Chinese TikTok in terms of intrinsic attributes, surface form, and deep content. The results show that in order to increase the number of fans of female-oriented accounts on Chinese TikTok, there are four variables of the intrinsic attribute with statistically significant differences, 13 variables of the surface form with statistically significant differences, and 9 variables of the deep content with statistically significant differences. Beauty-related content is not the best means to attract fans. It requires TikTok creators to get rid of stereotyped and conceptual female impressions, display the rich social roles of women, and build true, complete, and harmonious female images in innovative ways in accordance with the humanistic communication concept.

## Introduction

In recent years, China's short-video platforms have witnessed exponential growth. In just a few years, online short videos have become omnipresent and have reached almost every aspect of society. With the release of Kuaishou, Miaopai, TikTok, Volcano Video, and Nani Short Video in 2012, 2013, 2016, and 2018, respectively, short videos have become an indispensable part of social life in China. TikTok, a short-video app featuring video content between 15 and 60 s long, has become immensely popular around the world (Zeng et al., 2021). Montag et al. (2021) provided that insights from uses and gratification theory in the realm of TikTok are highlighted. Zhang (2021) pointed that TikTok could play the role of public supervision, dredge social contradictions, and alleviate social anxiety. According to *2020* TikTok Data Report (2021) released in January 2021, daily active users of TikTok have exceeded 600 million, and the average number of daily video searches has exceeded 400 million. In this context, the research enthusiasm for TikTok in academia is also increasing. On CNKI alone, which is a platform that provides various academic literature resources for readers at home and abroad, there are 1,902 literature studies on TikTok in 2018, 2019, and 2020. But most of them focus on the study of TikTok's operating mechanism and marketing strategy from the perspective of communicators. However, there is very few relevant research on the factors that influence the number of TikTok fans from the perspective of content. At present, there are a large number of female KOLs (Key Opinion Leaders) in short videos in China, and the empirical research and phenomenological interpretation of female TikTok accounts from the perspective of feminism are also lacking.

Traditional researches of feminism mainly focus on two topics: media image of women and woman's discourse. In terms of media image of women, Ma and Liang (2021) consider that some variety shows of women try to break the age limit, show women's diversified beauty and independent value, praise women's struggle, and try to build a more diversified and rich female media image. Tu and Wang (2021) found that the diversified image construction in the short video not only reflects women's situation and conveys women's power, but also deepens stereotypes and strengthens gender discrimination. In terms of woman's discourse, Li (2017) found that in the new media environment, the open expression, collision, and circulation of information are conducive to stimulate women's gender awareness. The low threshold use of We Media makes it possible for women to grasp the right to speak equally. However, the deep-rooted traditional culture, the operation power of capital, and the spread of consumer culture have conspired to dispel women's right to speak again. He (2018) thinks that the patriarchal system also extends to cyberspace. Cyberspace constantly compromises with men's economy, which hinders and distorts women's discourse expression and deepens women's stereotype.

Chen and Hu (2020) suggested that more females than males use the platform of TikTok. Regarding the influence of female content in consumption, Cai and Mao (2008) conducted a detailed and in-depth investigation from the perspectives of feminism, semiotics, and consumerism in their article “Female Beauty, Power and Consumption in Images: An Analysis of TV Beauty Pageant and TV Plastic Surgery Program.” It is found that the seemingly simple and happy transmission of female images is in fact a delicate lie created by the patriarchal culture by manipulating the media, which embodies the conspiracy between media and consumer industry (Su, 2016). How is this conspiracy accomplished? What factors affect the number of female TikTok fans? Ouyang and Liu (2013) believe that the technique of publishing self-media content, that is, the form of self-media, is an important variable that affects the number of self-media fans. It is true that due to intrinsic attributes, female-oriented accounts on Chinese TikTok can increase the number of fans by improving communication strategies, but is the content of the communication important? In other words, to what extent do the forms and content on We-Media affect the number of fans? This article examines the influence of the intrinsic attributes, the surface form, and deep content on the number of fans and attempts to construct an interactive relationship model between them.

## Research Design

### Research Methods

The main research method is content analysis, and the sampling method is a recommendation algorithm, which uses the user's browsing habits and behavior to push similar content to the user through a mathematical algorithm. The sample selection in this article is based on a random sampling recommendation algorithm, and 500 female-oriented accounts on Chinese TikTok are selected as the research samples. The data collection time is from the first TikTok post of each account to 0:00 on September 1, 2021. In this paper, SPSS23.0 software is used for statistics. The statistical methods used include independent sample *t*-test, correlation analysis, and linear regression analysis.

### Operational Definition of the Intrinsic Attribute

The intrinsic attribute of TikTok refers to the relatively fixed attribute of TikTok itself, which is generally the corresponding information displayed on the home page after TikTok is searched. Lin and Li (2015) divided the internal attributes of Weibo automobile brands into 11 variables in the article “Empirical Research on the Influencing Factors of Sina Weibo Automobile Brand Fans.” On this basis, combined with the characteristics of TikTok, this article subdivides TikTok's intrinsic attributes into five variables: “The number of days the account is opened,” “Emoji in the account name,” “Product display on the account homepage,” “Personal information on the account homepage,” and “Information of accounts on other platforms on the homepage,” as shown in Table 1.

**Table 1:** Description and coding standard of the intrinsic attribute-related variables.

**Index**	**Instruction**	**Coding standard**
The number of days to open TikTok	The number of days from the beginning of release of the first TikTok to 0 clock of September 1, 2021.	The number of days is recorded as 1, 2, 3……
Whether the nickname contains expression elements	A nickname is a name that an individual presents to the public according to her own preferences. It can include words, letters, symbols and expressions.	In the nickname, the one without expression is marked as 0, and the one with expression or only with expression is marked as 1.
Whether there is a commodity window display on the personal home page	Shop window is a kind of commodity sharing function of TikTok, which can be opened after self application and approval.	If there is no shop window, it is recorded as 0; if there is shop window, it is recorded as 1.
Whether there is a personal profile on the personal home page	Personal profile is a kind of personalized self introduction of TikTok. Users can edit personalized or comprehensive information in this column to make the public quickly understand or deepen their impression.	0 for no profile and 1 for profile.
Whether it is marked with other platform accounts	Mark other personal platform accounts in personal profile of TikTok, such as Microblog, Wechat, Little Red Book, IG and other account names.	If it is not marked, it will be marked as 0, and if it is marked, it will be marked as 1.

### Operational Definition of the Surface Form

Chen et al. (2013) extracted 13 variables related to the surface form of Weibo in the article “Comparative Study on the Expression of Mobile Weibo and Non-mobile Weibo: Taking Sina Weibo as an Example.” In this paper, 19 variables are added and adjusted as follows: “total number of TikTok posts,” “density of TikTok posts,” “number of posts concerning others,” “number of shares,” “density of shares,” “number of comments,” “density of comments,” “number of likes,” “density of likes,” “number of posts containing pictures,” “density of posts containing pictures,” “number of posts containing videos,” “density of posts containing videos,” “number of posts containing stickers,” “density of posts containing stickers,” “number of posts containing commodity links,” “density of posts containing commodity links,” “number of posts containing advertisements,” and “density of posts containing advertisements.” It is shown in Table 2.

**Table 2:** Description and coding standard of the surface form.

**Index**	**Instruction**
Total number of TikTok posts	The number of posts from the beginning of release of the first TikTok to 0 clock of September 1, 2021.
Density of TikTok posts	Total number of TikTok posts÷the number of days releasing TikTok
Number of TikTok concerning others	The number of TikTok concerning others from the beginning of release of the first TikTok to 0 clock of September 1, 2021.
Number of TikTok shared	The number of TikTok shared from the beginning of release of the first TikTok to 0 clock of September 1, 2021.
Density of TikTok shared	Total number of TikTok shared÷total number of TikTok posts
Number of TikTok commented	Number of TikTok commented from the beginning of release of the first TikTok to 0 clock of September 1, 2021.
Density of TikTok commented	Total number of TikTok commented÷total number of TikTok posts
Number of TikTok praised	Number of TikTok praised from the beginning of release of the first TikTok to 0 clock of September 1, 2021.
Density of TikTok praised	Total number of TikTok praised÷total number of TikTok posts
Number of TikTok including pictures	Total number of TikTok works created by users in the form of static pictures
Density of TikTok including pictures	Total number of TikTok works created by users in the form of static pictures÷total number of TikTok posts
Number of TikTok including videos	Total number of TikTok works created by users in the form of dynamic video
Density of TikTok including videos	Total number of TikTok works created by users in the form of dynamic video÷total number of TikTok posts
Number of TikTok including special effects for stickers	Total number of TikTok stickers or special effects used in TikTok works published by users
Density of TikTok including special effects for stickers	Total number of TikTok including special effects for stickers÷total number of TikTok posts
Number of TikTok commodity links	Total number of product links in TikTok works published by users
Density of TikTok commodity links	Total number of TikTok commodity links÷total number of TikTok posts
Number of TikTok advertisements	Total number of advertisements in TikTok works published by users
Density of TikTok advertisements	Total number of TikTok advertisements÷total number of TikTok posts

### Operational Definition of the Deep Content

TikTok User Portrait Report (2020) divides TikTok user preferences into 14 categories: music, creativity, dance, sketch, film and television, TikTok, beauty, food, lifestyle, car, mother and baby, game, two dimension world, and tourism. On this basis, this paper adjusted to 24 variables according to the actual situation: “number of joke posts,” “density of joke posts,” “number of emotion-related posts,” “density of emotion-related posts,” “number of fashion-related posts,” “density of fashion-related posts,” “number of beauty-related posts,” “density of beauty-related posts,” “number of lifestyle-related posts,” “density of lifestyle-related posts,” “number of posts related to appearance and figure,” “density of posts related to appearance and figure,” “number of hobby-related posts,” “density of hobby-related posts,” “number of posts for educational services promotion,” “density of posts for educational services promotion,” “number of posts related to maternal and child care,” “density of posts related to maternal and child care,” “number of posts related to make-up and styling,” “density of posts related to make-up and styling,” “number of posts related to talent exhibition,” “density of posts related to talent exhibition,” “number of posts related to celebrity gossip,” and “density of posts related to celebrity gossip.” It is shown in Table 3.

**Table 3:** Description and coding standard of the deep content.

**Index**	**Instruction**
Number of TikTok related to the subclass of jokes	Sitcoms, self created jokes, funny dialogue
Density of TikTok related to the subclass of jokes	Number of TikTok related to the subclass of jokes÷total number of TikTok posts
Number of TikTok related to emotional psychology	Number of TikTok related to the lovers' life, emotional advice and life perception
Density of TikTok related to emotional psychology	Number of TikTok related to emotional psychology÷total number of TikTok posts
Number of TikTok related to fashion	Clothing collocation, bag decoration collocation, dress shop display, style or design
Density of TikTok related to fashion	Number of TikTok related to fashion÷total number of TikTok posts
Number of TikTok related to beauty	Make up course, hairstyle course, skin care process
Density of TikTok related to beauty	Number of TikTok related to beauty÷total number of TikTok posts
Number of TikTok related to daily life	Daily life and work
Density of TikTok related to daily life	Number of TikTok related to daily life÷total number of TikTok posts
Number of TikTok related to appearance and figure	Personal appearance, partial facial features, photo
Density of TikTok related to appearance and figure	Number of TikTok related to appearance and figure÷total number of TikTok posts
Number of TikTok related to hobbies	Fitness, photography, games, tourism, pets, food
Density of TikTok related to hobbies	Number of TikTok related to hobbies÷total number of TikTok posts
Number of TikTok related to teaching promotion	Map repair skills, photo taking posture, life promotion, shop exploration
Density of TikTok related to teaching promotion	Number of TikTok related to teaching promotion÷total number of TikTok posts
Number of TikTok related to maternal and child care	Recording the baby's life, popular science and parenting knowledge
Density of TikTok related to maternal and child care	Number of TikTok related to maternal and child care÷total number of TikTok posts
Number of TikTok related to make-up and body styling	Using video clips to make a big change in personal image
Density of TikTok related to make-up and body styling	Number of TikTok related to make-up and styling÷total number of TikTok posts
Number of TikTok related to talent exhibition	Singing, dubbing, dancing
Density of TikTok related to talent exhibition	Number of TikTok related to talent exhibition÷total number of TikTok posts
Number of TikTok related to entertainment gossip	It refers to personal forwarding or editing of star shots, movie scenes, entertainment videos, etc.
Density of TikTok related to entertainment gossip	Number of TikTok related to entertainment gossip÷total number of TikTok posts

### Reliability and Validity

In content analysis, the factors affecting reliability and validity mainly come from systematic error and random error. The unscientific division of hesitation categories, the unclear definition of categories, and the experience limitations of coders themselves all belong to systematic errors. Systematic error is something we should try our best to avoid. According to Holsti's coefficient reliability, the acceptable reliability is required to be 0.9 or above (Yu, 2004). The reliability coefficient of the above-mentioned category construction in this paper was 0.913, which met the requirements. There is no evaluation formula for the validity of content analysis, but researchers need to self-examine or hire experts to evaluate the category construction. In this study, three experts in the fields of TikTok and feminism were employed to evaluate the category construction, which also met the basic requirements of validity.

## Data Results and Analysis

### The Influence of the Intrinsic Attribute on the Fans of Female TikTok

Among the five intrinsic attribute variables, there are four variables with statistically significant differences for the number of female TikTok fans: “The number of days the account is opened,” “Emoji in the account name,” “Product display on the account homepage,” and “Information of accounts on other platforms on the homepage.” The statistical methods involved include correlation analysis and independent sample *t*-test.

Correlation analysis was used to test the correlation between “number of days the account is opened” and “number of fans of female-oriented accounts on Chinese TikTok.” The results showed that *P* = 0.000, which was less than the significance level of 0.05, indicates that there was a correlation between the two. The correlation coefficient r is equal to 0.198^***^, indicating that there is a weak positive correlation between the two. That is to say, the earlier the account was open, the more fans of the account will attract. Since its launch in September 2016, TikTok has become a strong force in the short-video race. Because it is easy to use and accessible to all, even users who cannot edit videos professionally and cannot read much can produce and publish videos in a short time. As video production is becoming easier and more KOLs are emerging, a huge fan base was formed. Therefore, the earlier the account was open, the more fans of the account will attract.

Independent sample *t*-test was used to test whether there was any difference in the average number of female TikTok fans with “Emoji in the account name.” The results showed that concomitant probability was 0.000, which was less than the significance level of 0.05. The result shows that there is some difference in the average number of female TikTok fans with “Emoji in the account name.” The average number of fans of “accounts with emoji in the name” is 566,302.29, while the average number of fans of “accounts without emoji in the name” is 136,747.43. It means that the number of fans of “accounts with emoji in the name” is higher than that of “accounts without emoji in the name.” Marandro and Barker (1991) pointed out in non-verbal communication that non-verbal communication accounts for a large proportion of interpersonal communication, and it can provide important supplementary information such as role, emotion, voice and so on. Users add emoji in their account names, which enriches the diversity of nicknames and adds interest and vividness. The emoji in the name makes up for the limitation of words in conveying emotion and can attract fans' attention.

Independent sample *t*-test was used to test whether there was any difference in the average number of TikTok fans for accounts with “Product display on the account homepage.” The results showed that concomitant probability was 0.000, which was less than the significance level of 0.05. The result shows that there is some difference in the average number of TikTok fans for accounts with “Product display on the account homepage.” The average number of TikTok fans for accounts with “Product display on the account homepage” is 924,435.82, while the average number of TikTok fans for accounts without “Product display on the account homepage” is 121,361.22. It means that the number of TikTok fans for accounts with “Product display on the account homepage” is higher than that of accounts without “Product display on the account homepage.” Not everyone is qualified to open the window display function in the account homepage. One condition that needs to be met is that the number of users' fans should be ≥1,000. This function is usually activated by KOL (Key Opinion Leader) on TikTok. Li and Gao (2020) pointed out in the article “TikTok Advertising Marketing Strategy from the Perspective of Audience Psychology” that in the emotional marketing or internet celebrity activities of TikTok KOL, the audience will follow the crowd psychology, so as to promote the expansion of marketing flow. It also attracts the group that was not the target audience and then leads to the expansion of the number of ordinary fans. This study confirmed the conclusion.

Independent sample *t*-test was used to test whether there was any difference in the average number of TikTok fans for accounts with “information of accounts on other platforms on the homepage.” The results showed that concomitant probability was 0.000, which was less than the significance level of 0.05. The result shows that there is some difference in the average number of TikTok fans for accounts with “information of accounts on other platforms on the homepage.” The average number of TikTok fans for accounts with “information of accounts on other platforms on the homepage” is 814,038.64, while the average number of TikTok fans for accounts without “information of accounts on other platforms on the homepage” is 204,274.88. It means that the number of TikTok fans for accounts with “information of accounts on other platforms on the homepage” is higher than that of accounts without “information of accounts on other platforms on the homepage.” Liu and Wang (2018) in the article “Analysis of the Communication Mode of Opinion Leaders in Short Video App” think that KOLs on TikTok attract most of the fans of like mind around. Users use weak connections to spread content on the TikTok platform, thus forming an open circle for social purposes. Some KOLs on TikTok have a large follower base on other platforms. In order to increase fan stickiness, they often provide their accounts on other platforms on the TikTok platform. On the one hand, the newly opened TikTok account is attracting new fans, and on the other hand, it is also constantly channeling old fans from Weibo, Taobao, Xiaohongshu, Kuaishou, and other platforms, further expanding the fan base.

### The Influence of the Surface Form on the Fans of Female TikTok

In the correlation analysis, there were 13 variables with statistically significance (^*^p < 0.05, ^**^p < 0.01, and ^***^p < 0.001) for the number of fans of female-oriented accounts on Chinese TikTok: “total number of TikTok posts” (r = 0.352^***^), “number of shares” (r = 0.859^***^), “density of shares” (r = 0.347^***^), “number of comments” (r = 0.551^***^), “density of comments” (r = 0.590^**^), “number of likes” (r = 0.303^*^), “density of likes” (r = 0.320^***^), “density of posts including pictures” (r = −0.177^***^), “number of posts containing videos” (r = 0.385^***^), “density of posts including videos” (r = 0.177^***^), “number of posts containing stickers” (r = 0.254^**^), “number of posts containing advertisements” (r = 0.291^*^), and “density of TikTok advertisements” (r = 0.236^*^).

**Figure 1:**
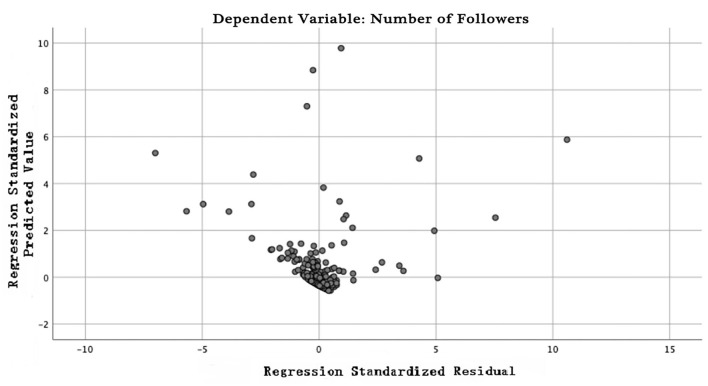
Scatter diagram of regression analysis effect of the surface form.

In this paper, linear regression analysis is used to test the number of fans of female-oriented accounts on Chinese TikTok and variables of the surface form. In order to avoid the multicollinearity problem, we use adjusted R-square to see whether we want to keep a variable in the model by looking at the increase or decrease of an independent variable. At last, 10 independent variables are used to conduct linear regression analysis on the number of fans as dependent variables. Ten independent variables include as follows: “total number of TikTok posts,” “number of TikTok concerning others,” “number of shares,” “number of comments,” “number of likes,” “number of posts containing pictures,” “number of posts containing videos,” “number of posts containing stickers,” “number of posts containing commodity links,” and “number of posts containing advertisements.” In this paper, linear regression analysis is used to test the surface form variables of female-oriented accounts on Chinese TikTok and the number of fans. At last, 10 independent variables are used to conduct linear regression analysis on the number of fans as dependent variables. The statistical results show that the correlation coefficient R is equal to 0.878, the judgment coefficient *R*^2^ is equal to 0.771, and the adjusted judgment coefficient *R*^−2^ is equal to 0.766, indicating that the sample regression effect is good. *P* is equal to 0.000 less than significant level 0.05, indicating that there is a linear regression relationship between independent variables and dependent variables, and six variables are included in the regression equation. The standardized regression equation is y = −0.085x_1_+ 0.789x_2_+ 0.111x_3_-0.052x_4_+ 0.114x_5_+ 0.131x_6_, in which y represents number of fans, x_1_ represents “total number of TikTok posts,” x_2_ represents “number of shares,” x_3_ represents “number of comments,” x_4_ represents “number of posts containing pictures,” x_5_ represents “number of posts containing stickers,” and x_6_ represents “number of posts containing advertisements.” That is to say, the less the “total number of TikTok posts” and “number of posts containing pictures,” at the same time the more the “number of shares,” “number of comments,” “number of posts containing pictures,” “number of posts containing stickers,” and “number of posts containing advertisements,” then the more the fans of female-oriented accounts on Chinese TikTok, as is shown in Table 4. The scatter diagram of regression analysis effect is shown in Figure 1.

**Table 4:** Linear regression test results for the number of TikTok and the number of fans.

**Index**	**Standardized regression coefficient**	**Sig**
Total number of TikTok posts	−0.085^**^	0.007
number of TikTok shared	0.789^***^	0.000
Number of TikTok commented	0.111^***^	0.000
Number of TikTok including pictures	−0.052^*^	0.024
Number of TikTok including special effects for stickers	0.114^***^	0.000
Number of TikTok advertisements	0.131^***^	0.000

* *p < 0.05*,

** *p < 0.01*,

*** *p < 0.001*.

In this paper, linear regression analysis is used to test the number of fans of female-oriented accounts on Chinese TikTok and the variables of surface form. Nine independent variables are used to conduct linear regression analysis on the density of fans as dependent variables, so as to avoid the multicollinearity problem. Nine independent variables include as follows: “density of TikTok posts,” “density of shares,” “density of comments,” “density of likes,” “density of posts containing pictures,” “density of posts containing videos,” “density of posts containing special effects for stickers,” “density of posts containing commodity links,” and “density of posts containing advertisements.” In this paper, linear regression analysis is used to test the surface form variables of female TikTok and the density of fans. Nine independent variables are used to conduct linear regression analysis on the number of fans of as dependent variables, so as to avoid the multicollinearity problem. Statistical results show that the correlation coefficient R is equal to 0.667, the judgment coefficient R^2^ is equal to 0.444, and the adjusted judgment coefficient *R*^−2^ is equal to 0.435, indicating that the sample regression effect is not good. P is equal to 0.587 above the significant level 0.05, indicating that there is not a linear regression relationship between independent variables and dependent variables.

### The Influence of the Deep Content on the Fans of Female TikTok

In the correlation analysis, there were nine variables with statistically significant differences for the number of female TikTok fans: “number of joke posts,” “density of emotion-related posts,” “number of emotion-related posts,” “density of lifestyle-related posts,” “number of posts related to appearance and figure,” “density of posts related to appearance and figure,” “number of posts related to make-up and styling,” “density of posts related to make-up and styling,” and “number of posts related to talent exhibition.” The specific results are shown in Table 5. From the value of the correlation coefficient, the largest coefficient is “number of posts related to make-up and styling.” The second is “number of sketch posts.” “Number of posts related to appearance and figure” just ranked sixth. This shows that for female-oriented accounts on Chinese TikTok, the most effective way to attract fans is not by merely relying on showing female bodies. Female-oriented accounts on Chinese TikTok, which can bring a sense of novelty, interaction, relaxation, and entertainment, have a stronger ability to attract fans.

**Table 5:** Results of correlation analysis between the deep content and the number of female TikTok fans.

**Index**	**Correlation analysis**	**Number of fans**
Number of TikTok related to the subclass of	Pearson correlation	0.346^***^
jokes	Sig (bilateral)	0.000
Density of TikTok related to the subclass of	Pearson correlation	0.185^***^
jokes	Sig (bilateral)	0.000
Number of TikTok related to emotional	Pearson correlation	0.211^**^
psychology	Sig (bilateral)	0.000
Density of TikTok related to emotional	Pearson correlation	0.078
psychology	Sig (bilateral)	0.081
Number of TikTok related to fashion	Pearson correlation	−0.021
	Sig (bilateral)	0.641
Density of TikTok related to fashion	Pearson correlation	−0.062
	Sig (bilateral)	0.169
Number of TikTok related to beauty	Pearson correlation	0.045
	Sig (bilateral)	0.316
Density of TikTok related to beauty	Pearson correlation	−0.002
	Sig (bilateral)	0.968
Number of TikTok related to daily life	Pearson correlation	0.038
	Sig (bilateral)	0.400
Density of TikTok related to daily life	Pearson correlation	−0.103^*^
	Sig (bilateral)	0.021
Number of TikTok related to appearance and	Pearson correlation	0.114^*^
figure	Sig (bilateral)	0.010
Density of TikTok related to appearance and	Pearson correlation	−0.092^*^
figure	Sig (bilateral)	0.039
Number of TikTok related to hobbies	Pearson correlation	−0.007
	Sig (bilateral)	0.882
Density of TikTok related to hobbies	Pearson correlation	−0.063
	Sig (bilateral)	0.159
Number of TikTok related to teaching	Pearson correlation	0.011
promotion	Sig (bilateral)	0.803
Density of TikTok related to teaching promotion	Pearson correlation	−0.023
	Sig (bilateral)	0.609
Number of TikTok related to maternal and child	Pearson correlation	−0.012
care	Sig (bilateral)	0.781
Density of TikTok related to maternal and child	Pearson correlation	−0.030
care	Sig (bilateral)	0.510
Number of TikTok related to make-up and	Pearson correlation	0.407^***^
styling	Sig (bilateral)	0.000
Density of TikTok related to make-up and	Pearson correlation	0.216^***^
styling	Sig (bilateral)	0.000
Number of TikTok related to talent exhibition	Pearson correlation	0.107^*^
	Sig (bilateral)	0.017
Density of TikTok related to talent exhibition	Pearson correlation	0.024
	Sig (bilateral)	0.585
Number of TikTok related to entertainment	Pearson correlation	0.037
gossip	Sig (bilateral)	0.406
Density of TikTok related to entertainment	Pearson correlation	0.007
gossip	Sig (bilateral)	0.871

* *p < 0.05*,

** *p < 0.01*,

*** *p < 0.001*.

In this paper, linear regression analysis is used to test the number of fans of female-oriented accounts on Chinese TikTok and the variables of deep content. At last, 12 independent variables are used to conduct linear regression analysis on the density of fans as dependent variables. Twelve independent variables include as follows: “number of sketch posts,” “number of emotion-related posts,” “number of fashion-related posts,” “number of beauty-related posts,” “number of lifestyle-related posts,” “number of posts related to appearance and figure,” “number of hobby-related posts,” “number of posts for educational services promotion,” “number of posts related to maternal and child care,” “number of posts related to make-up and styling,” “number of posts related to talent exhibition,” and “number of posts related to celebrity gossip.” In this paper, linear regression analysis is used to test the deep content variables of female-oriented accounts on Chinese TikTok and the number of fans. Twelve independent variables are used to conduct linear regression analysis on the number of fans of as dependent variables, so as to avoid the multicollinearity problem. Statistical results show that the correlation coefficient R is equal to 0.590, the judgment coefficient *R*^2^ is equal to 0.348, and the adjusted judgment coefficient *R*^−2^ is equal to 0.332, indicating that the sample regression effect is not good. *P* is equal to 0.217 above the significance level 0.05, indicating that there is not a linear regression relationship between independent variables and dependent variables.

In this paper, linear regression analysis is used to test the number of fans of female-oriented accounts on Chinese TikTok and the variables of deep content. At last, 12 independent variables are used to conduct linear regression analysis on the density of fans as dependent variables. Twelve independent variables include as follows: “density of emotion-related posts,” “density of emotion-related posts,” “density of posts related to fashion,” “density of posts related to beauty,” “density of lifestyle-related posts,” “density of posts related to appearance and figure,” “density of posts related to hobbies,” “density of posts for educational services promotion,” “density of TikTok related to maternal and child care,” “density of TikTok related to make-up and styling,” “density of posts related to talent exhibition,” and “density of posts related to celebrity gossip.” In this paper, linear regression analysis is used to test the deep content variables of female-oriented accounts on Chinese TikTok and the number of fans. Twelve independent variables are used to conduct linear regression analysis on the number of fans of as dependent variables, so as to avoid the multicollinearity problem. Statistical results show that the correlation coefficient R is equal to 0.316, the judgment coefficient *R*^2^ is equal to 0.100, and the adjusted judgment coefficient *R*^−2^ is equal to −0.079, indicating that the sample regression effect is not good. *P* is equal to 0.321 above the significant level 0.05, indicating that there is not a linear regression relationship between independent variables and dependent variables.

### Summary

To sum up, the data show that the more days the account is opened, the more fans for female TikTok. The number of female TikTok fans can be increased by the Emoji in the account name containing expression elements, product display on the account homepage, and information of accounts on other platforms on the homepage. The number of female fans affected by the number of TikTok shared, the density of TikTok commented, and the number of TikTok commented that ranked in the top three, indicating that the number of female TikTok fans is closely related to the degree of interaction with fans. Linear regression analysis shows that the less the total number of TikTok posts and the number of TikTok containing pictures and at the same time the more the number of TikTok shared, commented, sticker special-effected, and advertised, then the more the number of female TikTok fans. The largest correlation coefficient with the number of powder is “number of posts related to make-up and styling,” followed by “number of posts related to subclass of jokes,” indicating that it is very important for female TikTok content to produce novel effect, relaxation effect, and entertainment effect.

## Discussion

The results show that in order to increase the number of fans of female-oriented accounts on Chinese TikTok, there are four variables of the intrinsic attribute with statistically significant differences: “The number of days the account is opened,” “Emoji in the account name,” “Product display on the account homepage,” and “Information of accounts on other platforms on the homepage.” The earlier the account was open, the more fans of the account will attract. The number of fans of female-oriented accounts on Chinese TikTok can be increased by using Emoji in the account name, displaying commodities in the personal homepage and providing information of accounts on other platforms on the homepage.

There are 13 variables of the surface form with statistically significance: “total number of TikTok posts,” “number of shares,” “density of shares,” “number of comments,” “density of comments,” “number of likes,” “density of likes,” “density of posts containing pictures,” “number of posts containing videos,” “density of posts containing videos,” “number of posts containing stickers,” “number of posts containing advertisements,” and “density of posts containing advertisements.” Influence value ranked the top three as follows: “number of shares,” “density of comments,” and “number of comments.” The number of fans of female-oriented accounts on Chinese TikTok is closely related to the degree of fans' interaction. The fan base is inversely proportional to the “total number of TikTok posts” and “number of posts containing pictures” and is proportional to the “number of shares,” “number of comments,” “number of posts containing pictures,” “number of posts containing stickers,” and “number of posts containing advertisements.” It shows that an excessive number of fans can cause resistance in users. We should pay attention to the quality of TikTok, which can trigger the interaction of fans. The interactivity of pictures should be carefully used. The inclusion of advertising information in TikTok will not cause resistance in users, but helps to increase the number of fans.

There are nine variables of the deep content with statistically significant differences: “number of sketch posts,” “density of emotion-related posts,” “number of emotion-related posts,” “density of lifestyle-related posts,” “number of posts related to appearance and figure,” “density of posts related to appearance and figure,” “number of posts related to make-up and styling,” “density of posts related to make-up and styling,” and “number of posts related to talent exhibition.” The biggest correlation coefficient with the number of fans is “number of posts related to make-up and styling.” The second is “number of posts related to subclass of jokes.” It shows that it is very important for female-oriented accounts on TikTok to produce a sense of novelty, interaction, relaxation, and entertainment. Content that demonstrates the attractiveness of female bodies is not a very important way to attract fans.

Based on this, we believe that the influencing factors of the number of fans of female-oriented accounts on Chinese TikTok are closely related to the purpose of fans and the use of content. With the rapid development of China's social economy, the people are feeling an increasing amount of pressure in their lives and work. Female-oriented accounts on Chinese TikTok provide a channel to vent their pressure. People relax themselves in the funny and entertaining short videos. In communication, the short-video works of physical appearance can be called body communication or body narration as Yang (2017) described. Although Chinese TikTok do not lack female voice, the effect of sexual appeal has been fading with society developing. The strategy of attracting fans used female-oriented accounts on Chinese TikTok has changed from showing the external beauty of the body to focusing on the intellectual beauty of the inner needs. In the relationship between TikTok and the self-positioning of women, TikTok can be a guide, a maintainer of existing stereotypes, or a kitsch product. The biased female image on TikTok will lead to wrong value orientation and negative social impact, and it will also affect people's understanding and evaluation of modern women in Chinese society. With the advancement of Chinese women's status in politics, economy, and culture, women's self-awareness has been further awakened, and women's right to speak has expanded. Wang (2012) considered that feminists began to oppose the objectification of women, the “vase” positioning of women, and transformation of men's “vassal.” Yang (2020) found that short videos had attracted countless female users to show themselves on the platform and share their work, life, or family. The rise of female discourse and the spread of feminist thought are not to belittle men or surpass male hegemony, but to promote women's internal gender awakening and external gender dialog, so that they can have their own development path. Both women and the entire social group are calling for more harmonious and equal female images on TikTok.

Li (2013) considers that the differences between women and men are not innate and fixed. Accordingly, women's problems do not exist when human society appears. The emergence of this problem began with the change of women's labor mode, so the key to solve this problem lies in the change of labor mode and stereotype. Women's problem is a historical problem. The traditional division of labor in which women are responsible for housework and despised in social affairs has been questioned. Due to the changes in modern female labor style, Chinese women began to emerge in various fields. Their performance was no less than that of men. Similarly, women's living conditions and media impressions of vassal and enslavement cannot be permanent and unchangeable. Therefore, it requires TikTok creators to get rid of stereotyped and conceptual female impressions, display the rich social roles of women, and build true, complete, and harmonious female images in innovative ways in accordance with the humanistic communication concept.

## Data Availability Statement

The original contributions presented in the study are included in the article/supplementary material, further inquiries can be directed to the corresponding author.

## Author Contributions

YD analyzed data and wrote the manuscript. SL critically reviewed the manuscript. XZ was involved in formal analysis. All authors contributed to the article and approved the submitted version.

## Funding

This work was supported by the Fundamental Research Funds for Zhejiang Provincial Universities - Interdisciplinary Research (No. GB202103007).

## Conflict of Interest

The authors declare that the research was conducted in the absence of any commercial or financial relationships that could be construed as a potential conflict of interest.

## Publisher's Note

All claims expressed in this article are solely those of the authors and do not necessarily represent those of their affiliated organizations, or those of the publisher, the editors and the reviewers. Any product that may be evaluated in this article, or claim that may be made by its manufacturer, is not guaranteed or endorsed by the publisher.
